# Oak (*Quercus robur*) Associated Endophytic *Paenibacillus* sp. Promotes Poplar (*Populus* spp.) Root Growth In Vitro

**DOI:** 10.3390/microorganisms9061151

**Published:** 2021-05-27

**Authors:** Dorotėja Vaitiekūnaitė, Sigutė Kuusienė, Emilija Beniušytė

**Affiliations:** 1 Lithuanian Research Centre for Agriculture and Forestry, Laboratory of Forest Plant Biotechnology, Institute of Forestry, Liepu st. 1, Girionys, LT-53101 Šlienava, Lithuania; sigutekuus@gmail.com; 2 Faculty of Natural Sciences, Vytautas Magnus University, Universiteto st. 10, Akademija, LT-53361 Ringaudai, Lithuania; emilija.beniusyte@stud.vdu.it

**Keywords:** biofertilizer, *Paenibacillus*, *Populus*, *Quercus robur*, endophyte, bacteria, plant growth promotion, in vitro, microshoots

## Abstract

Soil fertilization is necessary for high-demand crop production in agriculture and forestry. Our current dependence on chemical fertilizers has significant harmful side effects. Biofertilization using microorganisms is a sustainable way to limit the need for chemical fertilizers in various enterprises. Most plant endophytic bacteria have thus far been unstudied for their plant growth promoting potential and hence present a novel niche for new biofertilizer strains. We isolated English oak (*Quercus robur*) endophytic bacteria and tested them for plant growth promoting traits (PGPTs) such as nitrogen fixation, phosphate mineralization/solubilization, siderophore and indole-3-acetic acid (IAA) production. We also investigated the effect the selected isolate had on poplar (*Populus* spp.) microshoot vegetative growth parameters in vitro. In total 48 bacterial strains were isolated, attributed to *Bacillus*, *Delftia*, *Paenibacillus*, *Pantoea* and *Pseudomonas* genera. All the isolates displayed at least three PGPTs, with 39.6% of the isolates displaying all five (all were *Pseudomonas* spp.) and 18.75% displaying four. Based on relative abundance, *Paenibacillus* sp. isolate was selected for the poplar microshoot inoculation study. The isolate had a significant positive effect on poplar microshoot root growth and development. Two tested poplar genotypes both had increased lateral root number and density, fresh and dry root biomass. Furthermore, one genotype had increased length and number of adventitious roots as well as a decrease in fresh aboveground biomass. The root enhancement was attributed to IAA production. We propose this isolate for further studies as a potential biofertilizer.

## 1. Introduction

Fertilization is a key aspect of continuous and efficient industrial production of various crop species in agriculture and forestry. However, our current dependence on chemical fertilizers is having a significant negative effect on ecology, soil and water quality, and human health, as well as having a global impact on carbon emissions [1,2,3,4,5,6,7]. Microorganisms (usually bacteria or fungi) used as biofertilizers can enhance the nutritional status of their host plants through a variety of mechanisms, thus enhancing crop yields [2,5]. These beneficial microbes are an eco-friendly way to reduce and perhaps even eliminate the need for chemical fertilization [1,2,3,5,8,9,10]. Due to their benefits, the demand for microbial-based biofertilizers and biocontrol agents is expected to rise in the future [2,4].

In open systems, plant growth may be limited by chemical insufficiencies or bioavailability of certain minerals. By inhabiting plant endospheres and rhizospheres, biofertilizer bacteria can enhance plant nutrient uptake and accessibility through nitrogen fixation, phosphate solubilization/mineralization, iron transport agent-siderophore production, etc. [11,12,13,14]. Additionally, bacteria can produce plant growth regulators, such as indole-3-acetic acid (IAA), which has been shown to positively affect plant growth and development [5]. Moreover, bacterial biofertilizers can help impede and/or limit the negative effects of both abiotic and biotic stressors [2,5]. Bacterial inoculants can be used for a single purpose, ex. N-fixation; however, more often bacteria can have several beneficial aspects and thus have a complex effect on their host [2,3,5,15,16].

Plant growth promoting bacteria (PGPB or PGPR, plant growth promoting rhizobacteria) can be used as a sustainable long-term solution for soil fertilization [3] or they can be used in tandem with chemical fertilizers, thus limiting the need for them, resulting in lower costs overall, a reduced negative impact on the ecosystem and a more sustainable end-product [3,8]. Moreover, it is likely that beneficial bacteria can create self-sustaining colonies in perennial plants, thus potentially extending their efficacy through multiple growing seasons [17,18,19,20,21].

Agriculturally important species have been shown to benefit from additional use of bacterial inoculants for sustainable crop production in previous studies [1,2,5,8]. In this case, the most thoroughly researched PGPB are the symbionts of legumes [10,15]. The effect of diazotroph inoculation on sugarcane has also been well documented [2,15]. Furthermore, works on other food species, such as wheat [22], apples [23], peanuts [24], tomatoes [25], etc., also show highly promising results.

Many biofertilizer trials have been focused on agricultural crops; however, studies suggest that trees and other woody plants are well suited for biofertilizer use. Several different bacterial inoculants have been successfully used both in pot trials and field studies on cedar [20], avocado [19], tea plant [16] and pine [26]. Furthermore, biofertilizers have been shown to work well concurrently with NPK (nitrogen, phosphorus and potassium) fertilizers as well as organic-matter-based fertilizer systems in several tree species: eucalyptus [7], orange trees [27], *Cordia africana*, *Croton macrostachyus*, *Erythrina brucei* and *Millettia ferruginea* [28], olive trees [29] and poplars [30]. Collectively these studies demonstrate that, at the very least, biofertilizers can limit the need for chemical fertilization without yield losses.

It is believed that in the future, one of the main goals in biofertilizer science will be the search for and thorough studies of new and/or yet unresearched microbe species [4,10]. In this aspect the endosphere of plants presents a novel niche, as so far all tested plants were shown to be inhabited by endophytic microorganisms [31]; however, just around 2% of all known plants were studied [32]. Oaks are promising as a source of yet-unstudied cultivable endophytic bacteria, as the genus is distributed widely throughout the Northern Hemisphere. English oaks (*Quercus robur*), specifically, are the model trees for the genus. They are essential hardwoods, known for providing habitat and food for a great variety of organisms in temperate forests, thus successfully thriving in multiple varied ecosystems. Oaks are also valuable for industrial forestry and other enterprises. Studies on endophytic oak bacteria are scarce. In the field of applicable endosymbionts, oak-associated fungal species are investigated as biocontrol agents for oak pathogen inhibition [33,34]; however, their cultivable bacterial endophytes are not thoroughly studied, nor is their use as biofertilizers.

Thus, the aim of this study was to investigate easily cultivable oak bacterial endophytes from oak phyllosphere and their plant growth promoting traits (PGPTs), as well as the effect selected bacteria may have on poplar (*Populus* spp.) microshoots in vitro for potential use as biofertilizers in future trials.

Five bacterial genera were isolated from the oak endosphere: *Bacillus*, *Delftia*, *Paenibacillus*, *Pantoea* and *Pseudomonas* (48 isolates in total). All the tested bacteria had at least three PGPTs. One IAA producing *Paenibacillus* sp. isolate was used for poplar inoculation study, demonstrating significant positive increases in lateral root number and density, fresh and dry root biomass, as well as a significant enhancement in adventitious root growth parameters.

## 2. Materials and Methods

### 2.1. Isolation and Identification

Seven field-grown English oaks were chosen for this study. Tree branches were gathered in late spring and force flushed in the lab to limit additional infection. Buds and leaves were used for endophyte isolation and sterilized using a modified surface sterilization method [35]. Leaves and buds were separated from the branches and first washed in a detergent solution (Tween 80) (1 drop/125 mL ddH_2_O (double distilled water)) for 5 min, then in 50% commercial bleach solution in ddH_2_O for 2 min. The third wash was done using 80% ethanol solution for 40 s. After each wash, samples were rinsed with ddH_2_O three times, 2 min each time.

To ensure that surface sterilization was effective, a modified method described by Sherling et al. [36] was used, whereupon explants were pressed against Woody plant medium (WPM) (Duchefa Biochemie, Haarlem, The Netherlands) [37] and observed for a month for microbial growth (+22 ± 1 °C). If no growth was observed, the sterilization was concluded to be a success and the bacteria isolated from these explants were considered to be endophytic.

After visualization, emerging morphologically different putative bacterial samples were selected and transferred from WPM onto Lysogeny broth (LB) agarized medium [38] (Duchefa Biochemie). Isolates were grown in a growth chamber at +22 ± 1 °C. Pure cultures were achieved by re-streaking the samples multiple times until single colonies appeared. As bacteria and yeast colonies look similar on agar medium, yeasts had to be separated out. To separate them, samples were observed under a microscope (1000× magnification) and distinguished on the basis of cell form and evidence of budding.

Bacterial isolates were sent to *Macrogen* sequencing center for DNA extraction and *16S rRNA* gene sequencing (Amsterdam, Netherlands). Universal 27F/800R and 518F/1492R primer set was used. Reverse primer sequences were transformed to forward complement sequences using open access *Chromas 2.6.6* software. Sequences were then edited using open access *Bioedit 7.2.5* software. National Center for Biotechnology Information’s (NCBI) *BLASTn* alignment tool was used to align sequences using standard parameters. Fragments were then matched with the NCBI database (BLAST Targeted Loci Nucleotide system) for *Bacteria* and *Archeae* (megablast) using ≥99% query coverage and ≥99% identity.

### 2.2. Plant Growth Promoting Traits

Screening for PGPTs was done in independent biological triplicates using fresh colonies each time. Bacteria were grown at +22 ± 1 °C. Isolates were tested for nitrogen fixation, phosphate solubilization and mineralization, siderophore and IAA production.

To screen for putative nitrogen fixation, as per Jasim et al. [39], nitrogen-free Jensen’s medium (Himedia, Mumbai, India) was used. The bacteria were spot inoculated on the medium (one sample per plate) and incubated for up to a week. Colony growth was evaluated and colonies with well-defined growth zones were considered putative diazotrophs.

Bacterial isolates were tested for phosphate solubilization and mineralization using an agar plate method [40]. Per Chen and Liu, two different phosphate sources were utilized: tricalcium phosphate—to test for inorganic phosphate solubilization (Pi)—and soy lecithin—to test for organic phosphate mineralization (Po). Bacteria were spot inoculated on the media and incubated for up to a week. The appearance of clear zones around the colonies indicated phosphate solubilization and/or mineralization.

To test for siderophore production, a modified Chromeazurol S (CAS) assay was used [41]. Prior to testing, all glassware was washed with 1mol/L HCl and then with ddH_2_O. Sterile CAS reagent was freshly prepared as described by Schwyn and Neilands before every test [42]. For the experiment, CAS reagent was mixed 1:9 with LB agarized medium. Bacterial isolates were then spot inoculated and incubated for up to a week. Orange/yellow zones around bacterial colonies were indicative of siderophore production.

To check if the bacteria were capable of tryptophan-dependent IAA production, a test with Salkowski reagent was used [43]. Bacteria were grown in LB broth (Duchefa Biochemie) enriched with tryptophan (0.15% *w/v*) in a thermalshaker (90 rpm) at 25 °C for 24 h in the dark. After that, 1.5 mL of this suspension was transferred to a microtube and centrifuged at 16300× g for 5 min. Then, 0.5 mL of supernatant was transferred to a cuvette and an equal amount of Salkowski reagent (1 mL 0.5M FeCl3 with 49 mL of 35% HClO4 *v/v*) was added. The cuvettes were incubated in the dark for 30 min. Afterwards, optical density was measured at 530 nm with T80+ UV/VIS spectrophotometer (PG Instruments LTD, Lutterworth, UK). Uninoculated tryptophan enriched LB broth and Salkowski reagent 1:1 was used as control. IAA production was estimated based on color change to red and checked using a standard curve.

### 2.3. Poplar Microshoot Inoculation

Based on relative abundance in all tested trees, one representative isolate was selected for this study. In vitro plant growth promoting effect of the selected isolate was tested on two model poplar tree genotypes, *Populus tremula* and *P. tremula* × *P. alba* hybrid. Both of these genotypes have been cultivated in vitro for at least 3 years. Poplars were chosen due to their status as model plants to study woody perennials as well as plant-microbiome interactions [44,45].

A modified method by Scherling et al. was used [36]. For both genotypes, the test was done under the same conditions, using WPM (additionally 4% gelrite, 2% sucrose). Two-month-old poplar microshoot culture was used, with 30 explants per group (divided into three independent replicates). For control groups microshoot leaves were removed and the stems were divided into 10 mm segments with 2–3 growing nodes each. Every segment was individually placed into ⌀20 mm glass tube with 5.25 mL of WPM. The tubes were capped and placed into a growth chamber at +22 ± 1 °C with 16/8 h day cycles.

For groups inoculated with bacteria, the bacterial inoculant was prepared first. A swab from a single bacterial colony was transferred to a fresh plate with LB medium and grown at +22 ± 1 °C overnight. The next day, prior to the transfer of microshoot segments, each tube was inoculated using an inoculation needle. Microshoot segments were then placed into the stab area. Otherwise, everything else was done in the same manner as with control groups.

After a 2-month incubation period, microshoot growth parameters were measured: shoot number and length, adventitious root number and length, width of the largest leaf, lateral root number, fresh and dry biomass (explants were dried for 2–3 h in a laboratory oven at 100 °C). Lateral root density was calculated. Data was analyzed using *Student’s* t-test and ANOVA (*Microsoft Excel*).

## 3. Results

In this study we assessed cultivable bacterial endophytes associated with English oak phyllosphere, their plant growth promoting traits and the effect selected isolate had on poplar microshoot vegetative growth parameters in vitro.

### 3.1. Isolation and Identification

After surface sterilization, putative bacteria created visible colonies on the medium surface or within the medium at the base of the explants within 2–3 weeks of incubation. In total, 48 viable bacterial samples were isolated.

Partial *16S rRNA* gene sequences from all 48 isolates were successfully obtained. Sequencing data is listed in Table 1. DNA sequencing yielded fragments that were 1324–1494 bp (base pairs) in length. The isolates were shown to belong to two different phyla of bacteria, *Proteobacteria* (52%) and *Firmicutes* (48%). Isolates were separated into five genera: *Bacillus*, *Delftia*, *Paenibacillus*, *Pantoea* and *Pseudomonas*. *Pseudomonas* spp. and *Paenibacillus* spp. were the most abundant, representing 39.6% and 37.5% of the total amount respectively, with *Bacillus* spp. representing 10.4%, and *Pantoea* spp. and *Delftia* spp. representing 6.25% of the total amount each.

### 3.2. Plant Growth Promoting Traits

**Figure 1:**
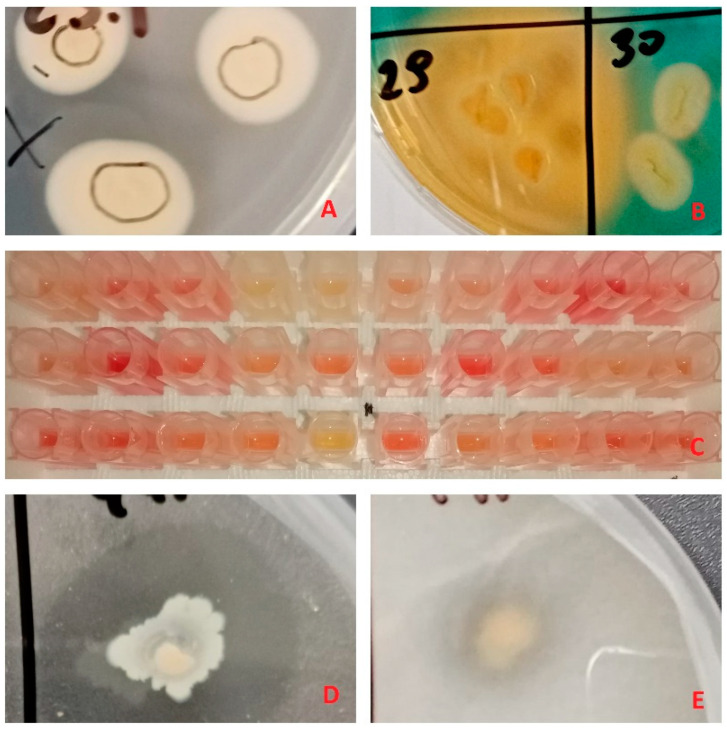
Examples of plant growth promoting trait tests: (**A**) bacterial growth on Jensen’s medium, indicating putative nitrogen fixation capabilities, (**B**) orange zones on Chromeazul S (CAS) medium, indicating siderophore production, (**C**) supernatant color change to red after addition of Salkowski reagent, indicating indole-3-acetic acid (IAA) production, (**D**,**E**) clear zones in insoluble phosphate enriched media, indicating phosphate mineralization and solubilization respectively.

All 48 isolates were screened for five PGPTs: nitrogen fixation, phosphate solubilization and mineralization, siderophore and IAA production (Table 1, Figure 1).

All isolates were capable of inorganic phosphate solubilization and IAA production. In the case of IAA production, amounts of produced IAA showed moderate alignment with bacterial genera, with *Pantoea* isolates producing relatively higher concentrations, *Paenibacillus* spp. and *Pseudomonas* spp. producing moderate amounts and *Bacillus* and *Delftia* isolates producing the least amount of IAA after 24 h incubation (Table 1).

Of all isolates, 95.8% showed organic phosphate mineralization. This ability was present in all the genera and absent in just two isolates, both of which showed high similarity to *Bacillus firmus* (>98% Identity), while 62.5% of the isolates were able to grow on nitrogen-free Jensen’s medium, suggesting atmospheric nitrogen fixation capabilities. The only isolates that failed to display any growth were from the *Paenibacillus* genus, and 39.6% of the isolates showed positive results for siderophore production. All of these were from the *Pseudomonas* genus.

All the isolates displayed at least three PGPTs, with 39.6% of the isolates displaying all five (all were *Pseudomonas* spp.) and 18.75% displaying four.

The representative isolate used in later studies was able to produce IAA from tryptophan, on average at 27 ± 0.002 µg/mL after 24 h incubation. It tested positive for phosphate solubilization and mineralization and tested negative for nitrogen fixation and siderophore production.

### 3.3. Poplar microshoot inoculation

Of all isolates, 37.5% had the same colony morphology after a 2-day incubation: colonies were circular, off white, with a grey bull’s eye, mucous, cells were rod-shaped, gram-negative. Partial *16S rRNA* gene sequencing results showed that all the isolates with this morphotype belong to genus *Paenibacillus*, and that almost all isolates in this group were closely related to *Paenibacillus tundrae* (NR_044525.1). Moreover, bacteria with this morphotype were found in 6 out of 7 trees tested. Based on this information, we selected one isolate (*P. tundrae* % Identity—99.39%, Query coverage—99%) from this group at random for poplar microshoot inoculation study.

**Figure 2:**
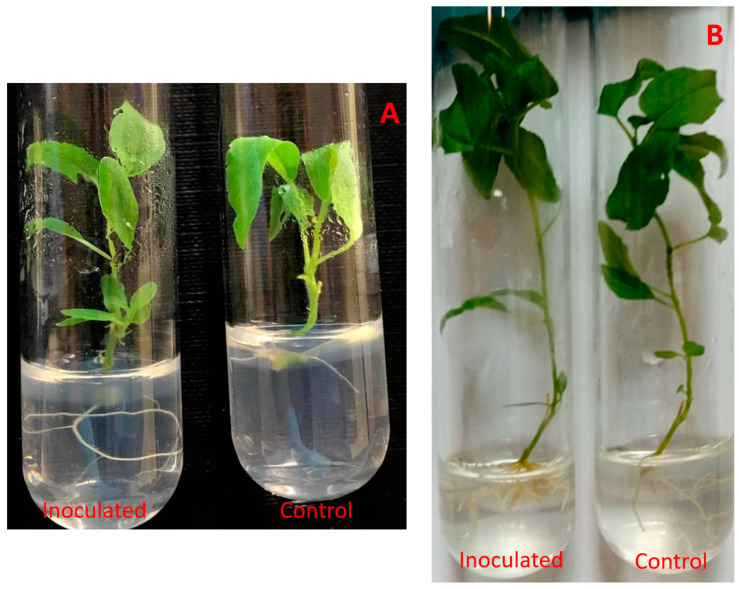
*Paenibacillus* sp. inoculated *P. tremula* microshoots after 2–3 weeks (**A**) and after 2 months (**B**) of incubation.

After a 2-month incubation, inoculated poplar microshoots had visible differences in regard to their respective control groups (Figure 2).

**Figure 3:**
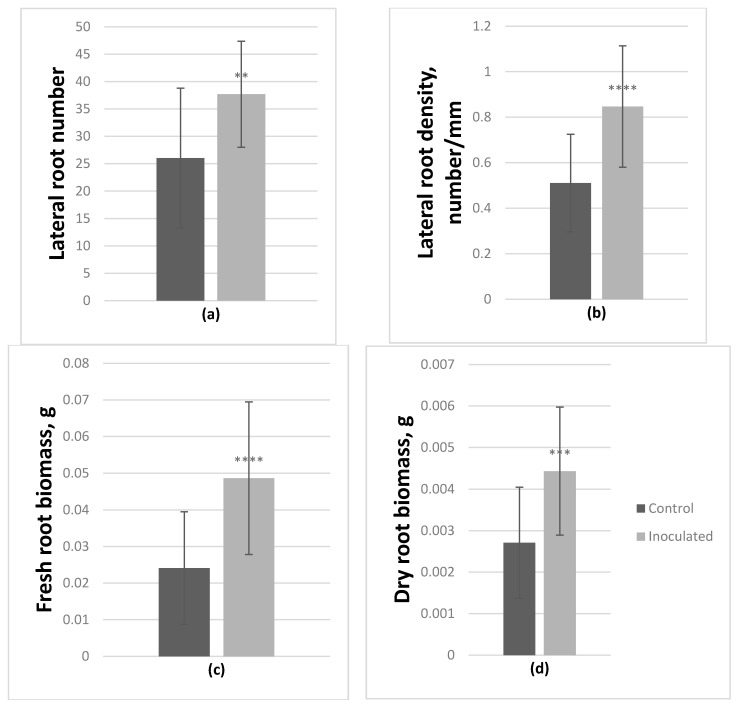
Effect of *Paenibacillus* sp. inoculation on *P. tremula* microshoots in vitro after 2-month incubation. On average lateral root number (**a**) and density (**b**) increased by 44.7% and 66% respectively, fresh root biomass (**c**) increased by 101.9% and dry root biomass (**d**) by 63.6% (average from 30 explants ± SD, **—*p* ≤ 0.01, ***—*p* ≤ 0.001, ****—*p* ≤ 0.0001).

Inoculation with *Paenibacillus* sp. had a significant positive effect on the growth of both genotypes (Table 2), which was most noticeable in the root systems. Based on ANOVA, on average, in inoculated *P. tremula* microshoots lateral root number significantly increased by 44.7%, lateral root density by 66%, fresh and dry root biomass by 101.9% and 63.6% respectively (Figure 3).

**Figure 4:**
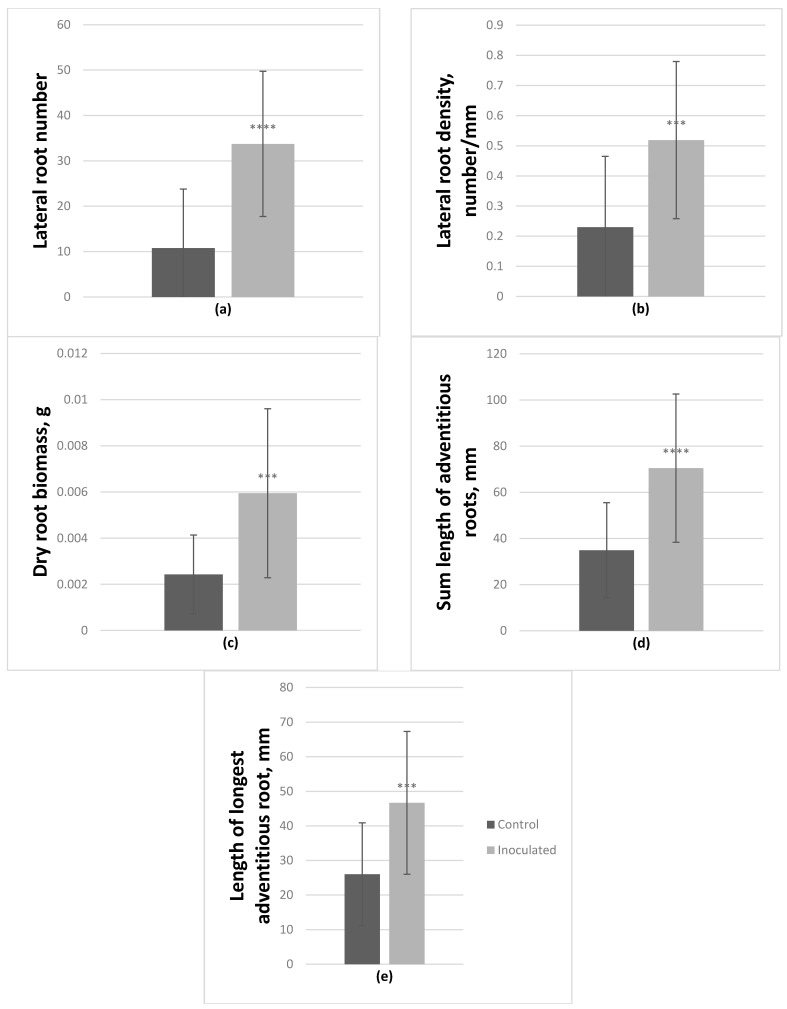
Effect of *Paenibacillus* sp. inoculation on *P. tremula* × *P. alba* microshoots in vitro after 2-month incubation. On average lateral root number (**a**) and density (**b**) increased by 213.7% and 125.6% respectively, dry root biomass (**c**) increased by 144.8%. Adventitious roots were also affected, on average their sum length (**d**) increased by 102% and the number of adventitious roots (**e**) increased by 65% (average from 30 explants ± SD, *** *p* ≤ 0.001, **** *p* ≤ 0.0001).

On average, *P. tremula* × *P. alba* hybrid microshoots also had significantly positively affected lateral root number and density; they increased by 213.7% and 125.6% respectively. Fresh and dry root biomass increased by 197.1% and 144.8% respectively. Inoculated microshoots also had longer adventitious roots by 102% (sum length), and the length of the longest adventitious root increased by 79.5% (Figure 4); the number of these roots was also enhanced by 65%. In this genotype, inoculation had a negative effect on fresh aboveground biomass, it decreased by 25%. 

## 4. Discussion

Obtained sequencing results showed that the isolated bacteria belong to two phyla, *Firmicutes* and *Proteobacteria*, in equal amounts. This coincides with other studies, that show that both of these phyla are often found in various woody plant hosts, accountable for much of the diversity of endophytic bacteria [5,35,46,47,48]. These two bacterial phyla were also found in two metabarcoding studies of English oak endophytes [49,50]. All the genera, except *Delftia* and *Pantoea*, found in this study, were also previously isolated from English oaks [51,52].

In our study bacterial isolates were categorized into 5 genera: *Bacillus*, *Delftia*, *Paenibacillus*, *Pantoea* and *Pseudomonas*, with *Paenibacillus* spp. and *Pseudomonas* spp. representing the bulk amount. Thus far, all 5 genera were isolated from several woody perennials [23,48,53,54,55,56,57,58,59,60,61]. Moreover, genera *Bacillus*, *Delftia*, *Pantoea* and *Pseudomonas* all have been shown to possess multiple PGPTs in earlier studies [16,39,40,62,63,64,65].

The representative isolate selected for poplar inoculation experiment was found to be closely related to *Paenibacillus tundrae*, a relatively novel species, first isolated from the soil in North America [66]. While, to our knowledge, this species was never sourced from trees, it has been shown to inhabit barley [67].

Collectively *Paenibacillus* are known as benign inhabitants of plant rhizospheres [67]. Thus far some have been isolated from trees such as poplar, larch and spruce [58], ash [54], Scots pine and rowan [55], western red cedar and lodgepole pine [56], coffee tree [59] and tea shrub [48].

Previously *Paenibacillus* spp. have been tested for plant growth promoting capabilities, mostly in agricultural species, showing promising results [24,68]. Furthermore, a nitrogen fixing *Paenibacillus* isolate (closely related to *P. humicus*) was previously tested on poplar microshoots. It had a significant effect on plant metabolic signature; additionally, it enhanced microshoot root length and number [36,58]. In our study, poplar microshoots were positively affected as well. This was especially evident in the root systems, where there was a significant increase in biomass, lateral root number and density in both tested genotypes.

Furthermore, while most inoculation effects were of similar nature in both tested genotypes, the hybrid poplar additionally had increased adventitious root length and number as well as decreased fresh aboveground biomass. Since no corresponding results were observed in *P. tremula* microshoots or dry aboveground biomass of the hybrid and hybrid poplars are known to grow faster than *P. tremula* [69,70], we attributed this decrease to nutrient and water depletion due to microshoot growth and feeding of the bacteria [71,72]. However, to test this hypothesis, further research is required.

The inoculant in our study tested positive for IAA production. Based on the results of earlier research and the biochemical properties of this plant growth regulator, it is likely that the effect on root growth in our in vitro study was instigated by IAA [5,73]. IAA is an auxin, which have been linked to promotion in root formation, due to enhanced stimulation of cell division, elongation and differentiation. Endophytic IAA production facilitates root system growth, which in due course boosts nutrient and water uptake [1,5,15,74,75]. Bal et al. reported that IAA producing *Paenibacillus* had had a positive effect on root growth as well. Inoculated rice seedlings demonstrated an increase in root length, dry and fresh root biomass, as well as shoot length and both dry and fresh shoot biomass, with a positive correlation between root elongation and IAA production [76]. Similar studies with IAA producing bacteria in other species, showed comparable results [77,78,79].

Moreover, trials with prototype biofertilizers of IAA producing *Paenibacillus* sp. demonstrated a positive effect not only in in vitro trials, but in pot trials as well. Inoculated mung beans showed a positive increase in dry biomass (17%) and sapling length (28%) (*p* < 0.05) [68].

## 5. Conclusions

Thus, in conclusion, we report that five different bacterial endophyte genera, *Bacillus*, *Delftia*, *Paenibacillus*, *Pantoea* and *Pseudomonas*, were isolated from English oak phyllosphere. All isolates were tested for nitrogen fixation, phosphate solubilization/mineralization, IAA and siderophore production. All tested bacteria had at least three of these traits. One isolate, closely related to *Paenibacillus tundrae*, was used in a poplar microshoot inoculation study. The study revealed that root growth of the inoculated plants was significantly increased. Lateral root density, number, fresh and dry root biomass were affected in both tested genotypes. Additionally, one of the genotypes tested had increased adventitious root growth. This root growth enhancement was attributed to IAA production by the isolate. We propose that, after further research, this isolate could potentially be used as a biofertilizer in forestry enterprises.

## Figures and Tables

**Table 1:** Sequence analysis of the *16S rRNA* gene and plant growth promoting properties of the 48 bacterial endophytes isolated from *Q. robur* phyllosphere (highlighted—isolate chosen for further inoculation study).

Sequencing Analysis				Plant Growth Promoting Traits					
Source Tree Designation	Fragment Length, bp	Closest NCBI Match, Accession no.	% Identity	Query Coverage, %	Siderophore Production	Phosphate Solubilization/Mineralization		Nitrogen Fixation	IAA Production, µg/mL
						Pi	Po		
4	1464	*Delftia lacustris* 332, NR_116495.1	99.86	100	-	+	+	+	16.36
1	1477	*Paenibacillus tundrae* A10b, NR_044525.1	99.32	99	-	+	+	-	25
3	1486	*Paenibacillus tundrae* A10b, NR_044525.1	99.53	99	-	+	+	-	29.2
I1	1483	*Bacillus aryabhattai* B8W22, NR_115953.1	99.93	99	-	+	+	+	7.4
I1	1483	*Bacillus aryabhattai* B8W22, NR_115953.1	99.80	99	-	+	+	+	13.6
2	1469	*Pseudomonas brenneri* CFML 97-391, NR_025103.1	99.86	99	+	+	+	+	17.5
I2	1462	*Pseudomonas azotoformans* NBRC 12693, NR_113600.1	99.73	99	+	+	+	+	17.5
3	1483	*Paenibacillus tundrae* A10b, NR_044525.1	99.46	100	-	+	+	-	27.2
1	1395	*Pantoea agglomerans* DSM 3493, NR_041978.1	99.64	100	-	+	+	+	50
I2	1486	*Paenibacillus tundrae* A10b, NR_044525.1	99.39	99	-	+	+	-	27.22
2	1473	*Pseudomonas proteolytica* CMS 64, NR_025588.1	99.59	99	+	+	+	+	13.2
1	1411	*Pantoea agglomerans* DSM 3493, NR_041978.1	99.65	100	-	+	+	+	67.6
I2	1485	*Paenibacillus tundrae* A10b, NR_044525.1	99.53	99	-	+	+	-	28.4
1	1461	*Pseudomonas azotoformans* NBRC 12693, NR_113600.1	99.66	99	+	+	+	+	18.2
1	1470	*Pseudomonas azotoformans* NBRC 12693, NR_113600.1	99.66	99	+	+	+	+	19.2
1	1488	*Paenibacillus tundrae* A10b, NR_044525.1	99.46	99	-	+	+	-	25.6
1	1459	*Pantoea agglomerans* DSM 3493, NR_041978.1	99.97	99	-	+	+	+	41.7
**2**	**1484**	***Paenibacillus tundrae* A10b, NR_044525.1**	**99.39**	**99**	**-**	**+**	**+**	**-**	**27**
1	1469	*Pseudomonas azotoformans* NBRC 12693, NR_113600.1	99.66	99	+	+	+	+	16.4
2	1472	*Pseudomonas azotoformans* NBRC 12693, NR_113600.1	99.73	99	+	+	+	+	18.3
I1	1486	*Paenibacillus tundrae* A10b, NR_044525.1	99.33	99	-	+	+	-	31.6
3	1490	*Paenibacillus tundrae* A10b, NR_044525.1	99.39	99	-	+	+	-	26.9
3	1467	*Pseudomonas azotoformans* NBRC 12693, NR_113600.1	99.73	99	+	+	+	+	19.2
1	1469	*Pseudomonas azotoformans* NBRC 12693, NR_113600.1	99.66	99	+	+	+	+	17
S2	1492	*Bacillus aryabhattai* B8W22, NR_115953.1	99.80	99	-	+	+	+	8.8
S2	1481	*Paenibacillus tundrae* A10b, NR_044525.1	99.53	99	-	+	+	-	25.2
3	1474	*Pseudomonas proteolytica* CMS 64, NR_025588.1	99.66	99	+	+	+	+	17
3	1472	*Pseudomonas azotoformans* NBRC 12693, NR_113600.1	99.73	99	+	+	+	+	17.5
3	1494	*Paenibacillus tundrae* A10b, NR_044525.1	99.53	99	-	+	+	-	22.4
3	1487	*Paenibacillus tundrae* A10b, NR_044525.1	99.53	99	-	+	+	-	26.4
3	1324	*Pseudomonas paralactis* DSM 29164, NR_156987.1	99.85	100	+	+	+	+	17.6
3	1354	*Paenibacillus tundrae* A10b, NR_044525.1	99.34	99	-	+	+	-	25.02
1	1466	*Pseudomonas azotoformans* NBRC 12693, NR_113600.1	99.73	99	+	+	+	+	16.4
1	1454	*Pseudomonas azotoformans* NBRC 12693, NR_113600.1	99.72	99	+	+	+	+	11.6
2	1467	*Pseudomonas azotoformans* NBRC 12693, NR_113600.1	99.73	99	+	+	+	+	17.5
2	1460	*Pseudomonas azotoformans* NBRC 12693, NR_113600.1	99.79	99	+	+	+	+	15
3	1487	*Paenibacillus tundrae* A10b, NR_044525.1	99.53	100	-	+	+	-	25
3	1475	*Bacillus firmus* NBRC 15306, NR_112635.1	98.17	100	-	+	-	+	13.2
1	1465	*Pseudomonas azotoformans* NBRC 12693, NR_113600.1	99.66	99	+	+	+	+	24.4
I2	1326	*Paenibacillus oceanisediminis* L10, NR_118217.1	98.93	99	-	+	+	-	25.04
I2	1484	*Paenibacillus tundrae* A10b, NR_044525.1	99.46	99	-	+	+	-	21.08
4	1339	*Delftia lacustris* 332, NR_116495.1	99.85	99	-	+	+	+	15.2
3	1493	*Pseudomonas azotoformans* NBRC 12693, NR_113600.1	98.18	99	+	+	+	+	18.16
3	1483	*Paenibacillus tundrae* A10b, NR_044525.1	99.46	100	-	+	+	-	30
3	1483	*Bacillus firmus* NBRC 15306, NR_112635.1	98.92	99	-	+	-	+	11.2
4	1468	*Pseudomonas azotoformans* NBRC 12693, NR_113600.1	99.79	99	+	+	+	+	27
4	1489	*Delftia lacustris* 332, NR_116495.1	98.39	100	-	+	+	+	17
I2	1490	*Paenibacillus tundrae* A10b, NR_044525.1	99.60	99	-	+	+	-	25

**Table 2:** Microshoot growth parameter measurements taken after 2 months of incubation and their statistical analysis data.

Averages ± SD of:	Shoot Length, mm	Shoot Number	Largest Leaf Width, mm	Number of Adventitious Roots	Number of Lateral Roots	Sum Length of Adventitious Roots, mm	Longest Adventitious Root, mm	Fresh Aboveground Biomass, g	Fresh Root Biomass, g	Dry Aboveground Biomass, g	Dry Root Biomass, g	Lateral Root Density, Number/mm
Uninoculated *P. tremula*	43.3 ± 9.2	1.2 ± 0.5	9.6 ± 2.4	3.6 ± 1.4	26.0 ± 12.7	50.9 ± 18.1	25.1 ± 10.9	0.065 ± 0.024	0.024 ± 0.015	0.013 ± 0.005	0.003 ± 0.001	0.51 ± 0.21
Inoculated *P. tremula*	43.5 ± 6.2	1 ± 0.0	11.3 ± 1.4	4 ± 1.1	37.7 ± 9.7 (↑44.7%)	47.6 ± 16.4	18.9 ± 7.7	0.075 ± 0.012	0.049 ± 0.021 (↑101.9%)	0.015 ± 0.002	0.004 ± 0.002 (↑63.6%)	0.85 ± 0.27 (↑66%)
Student’s t-test	NS	NS	**	NS	**	NS	*	NS	****	*	***	****
ANOVA	NS	NS	NS	NS	F (11.3), **	NS	NS	NS	F (19.4), ****	NS	F (15.4), ***	F (20.1), ****
Uninoculated *P. tremula* × *P. alba*	48.2 ± 14.2	1.0 ± 0.2	9.8 ± 2.3	1.6 ± 0.6	10.8 ± 13.0	34.9 ± 20.6	26 ± 14.9	0.157 ± 0.05	0.018 ± 0.021	0.028 ± 0.009	0.002 ± 0.002	0.23 ± 0.23
Inoculated *P. tremula* × *P. alba*	39.1 ± 12.1	1.0 ± 0.2	11.3 ± 2.8	2.7 ± 1.4 (↑65%)	33.7 ± 16.0 (↑213.7%)	70.5 ± 32.1 (↑102%)	46.7 ± 20.7 (↑79.5%)	0.118 ± 0.029 (↓25%)	0.053 ± 0.043 (↑197.1%)	0.022 ± 0.007	0.006 ± 0.004 (↑144.8%)	0.52 ± 0.26 (↑125.6%)
Student’s t-test	NS	NS	NS	***	****	***	***	**	**	*	***	***
ANOVA	NS	NS	NS	F(11.7), **	F(27.4), ****	F(19.5), ****	F(14.7), ***	F(9.9), **	F(12.2), **	NS	F(17.1), ***	F(15.0), ***

NS-nonsignificant. *p* > 0.05, *—*p* ≤ 0.05, **—*p* ≤ 0.01, ***—*p* ≤ 0.001, ****—*p* ≤ 0.0001.

## Data Availability

Detailed data concerning this study is available upon request.
